# Kaposi Sarcoma Herpesvirus (KSHV) Latency-Associated Nuclear Antigen (LANA) recruits components of the MRN (Mre11-Rad50-NBS1) repair complex to modulate an innate immune signaling pathway and viral latency

**DOI:** 10.1371/journal.ppat.1006335

**Published:** 2017-04-21

**Authors:** Giuseppe Mariggiò, Sandra Koch, Guigen Zhang, Magdalena Weidner-Glunde, Jessica Rückert, Semra Kati, Susann Santag, Thomas F. Schulz

**Affiliations:** 1 Institute of Virology, Hannover Medical School, Hannover, Germany; 2 German Centre for Infection Research, Hannover-Braunschweig Site, Germany; University of Pennsylvania Medical School, UNITED STATES

## Abstract

Kaposi Sarcoma Herpesvirus (KSHV), a *γ2-herpesvirus* and class 1 carcinogen, is responsible for at least three human malignancies: Kaposi Sarcoma (KS), Primary Effusion Lymphoma (PEL) and Multicentric Castleman’s Disease (MCD). Its major nuclear latency protein, LANA, is indispensable for the maintenance and replication of latent viral DNA in infected cells. Although LANA is mainly a nuclear protein, cytoplasmic isoforms of LANA exist and can act as antagonists of the cytoplasmic DNA sensor, cGAS. Here, we show that cytosolic LANA also recruits members of the MRN (Mre11-Rad50-NBS1) repair complex in the cytosol and thereby inhibits their recently reported role in the sensing of cytoplasmic DNA and activation of the NF-κB pathway. Inhibition of NF-κB activation by cytoplasmic LANA is accompanied by increased lytic replication in KSHV-infected cells, suggesting that MRN-dependent NF-κB activation contributes to KSHV latency. Cytoplasmic LANA may therefore support the activation of KSHV lytic replication in part by counteracting the activation of NF-κB in response to cytoplasmic DNA. This would complement the recently described role of cytoplasmic LANA in blocking an interferon response triggered by cGAS and thereby promoting lytic reactivation. Our findings highlight a second point at which cytoplasmic LANA interferes with the innate immune response, as well as the importance of the recently discovered role of cytoplasmic MRN complex members as innate sensors of cytoplasmic DNA for the control of KSHV replication.

## Author summary

KSHV latency-associated nuclear antigen, LANA, is essential for the replication of latent viral episomes, their segregation to daughter cells and overcoming the p53-dependent cell cycle block induced by an activated DNA damage response. In addition, cytoplasmic forms of LANA have been shown to modulate cGAS-dependent innate immune response. The findings presented in this report extend this role of cytoplasmic LANA to an innate immune response that is linked to the repair of double strand DNA breaks, thus reinforcing the importance of LANA as an antagonist of the innate immune response.

## Introduction

Kaposi Sarcoma Herpesvirus (KSHV or HHV-8, Human Herpesvirus 8), a *γ2-herpesvirus* or *Rhadinovirus* categorized as a class 1 carcinogen by the World Health Organization (WHO) [1–3], is responsible for Kaposi’s Sarcoma (KS), the most common cancer among HIV infected individuals (epidemic KS) and among men in Sub-Saharan African countries. KSHV is also the cause of two other rare lymphoproliferative disorders, namely Primary Effusion Lymphoma (PEL) and Multicentric Castleman’s Disease (MCD) [4,5]. Like other herpesviruses, KSHV can establish a lifelong latent infection and exhibits a biphasic life cycle consisting of latency, the default state, in which only few viral proteins are expressed, and lytic replication, which leads to the production of new virions and the death of the host cells [6]. The KSHV major latent protein, LANA (Latency-Associated Nuclear Antigen) is expressed in all infected KS, PEL or MCD cells [6–9]. In the prototypic BC1 strain [10], LANA is a protein of 1162 amino acids and consists of three main domains: the amino-terminal domain, the internal repeat (IR) domain and the carboxy-terminal domain. The N-terminal nuclear localization signal (NLS) is responsible for the nuclear localization of LANA, and is positioned near the chromatin binding domain (CBD) that tethers LANA to histones on cellular chromosomes during mitosis [11–13]. LANA can also bind to the viral latent origin of replication in the terminal repeats (TR) of the KSHV genome by means of a specific DNA-binding domain at its C-terminal end. LANA plays an essential role in latent viral DNA replication and episome maintenance as well as transcription regulation and interaction with crucial cellular factors [6,7,14–24]. More recently, non-canonical LANA isoforms have been identified [25,26]. These include truncated protein isoforms that originate from alternative start codons within the LANA N-terminal domain and therefore lack the NLS and as a consequence are located in the cytoplasm. We could recently show that such cytoplasmic LANA variants promote KSHV lytic reactivation by inhibiting the cGAS-STING-mediated activation of type I interferon response [27], which is triggered by cytosolic viral DNA; they may thus act as antagonists of full-length LANA, which is required for latent replication and partitioning of viral episomes to daughter cells in mitosis. LANA binds to cGAS, as identified by mass spectrometry (MS) and immunoprecipitation [27]. The same MS analysis yielded several other new putative LANA-binding proteins, among them several DNA Damage Repair/Response (DDR) proteins [27]. Here, we focus on the interaction of LANA with the MRN (Mre11-Rad50-NBS1) complex, an important sensor of double-strand DNA breaks (DSBs) that is responsible for the detection of DNA damage and the activation of the repair cascade [28,29]. It is well established that viruses evolved ways to exploit the cellular DDR machinery to their own benefit, such as the replication of viral DNA [30–41]. Moreover, it has also been shown that KSHV *de novo* infection, as well as lytic reactivation from latency, trigger the DDR response [32,33,38]. Furthermore, LANA has been reported to interact with Chk2 in order to dysregulate the cell cycle during the viral latency [18].

Recent studies have suggested that the ability of the MRN complex to sense damaged DNA also plays a role during the innate immune response to foreign DNA [42–44]. In this context, cytoplasmic Rad50 and Mre11, together with CARD9, sense cytoplasmic DNA and activate the NF-κB pathway [42]. We show here that cytoplasmic LANA isoforms recruit Rad50 and Mre11 in the cytosol and thereby interfere with the activation of the NF-κB cascade induced by transfected DNA, as well as KSHV reactivation from latency. These observations point to yet another antiviral mechanism inhibited by cytosolic LANA isoforms, and highlight the importance of the sensing of cytosolic DNA by the MRN complex in the context of innate immunity against viral infection.

## Results

### KSHV LANA recruits MRN (Mre11-Rad50-NBS1) complex proteins in KSHV-infected B cells

We recently reported the identification of several novel cellular KSHV LANA-interacting proteins by mass spectrometry in the KSHV infected PEL-derived cell line, BCBL-1 [27]. Among them were several cellular DNA damage response (DDR) proteins, including proteins involved in double-strand breaks (DSBs) recognition and repair (Rad50, Mre11, MDC1), mismatch repair (MSH2), and nucleotide excision repair (XPC, HR23B) [27]. Previous reports already point to a link between KSHV infection and DDR activation [15,16,18,23,45–49]. In this study, we focused on the binding of LANA to Rad50 and Mre11. Together with NBS1, Rad50 and Mre11 form the MRN (Mre11-Rad50-NBS1) complex, which is the upstream activator of the DSBs repair pathway, and is also involved in the replication of several DNA viruses [30,32,34–36,40,50]. Therefore, we proceeded to elucidate further the interaction between KSHV LANA and MRN complex components.

**Fig 1:**
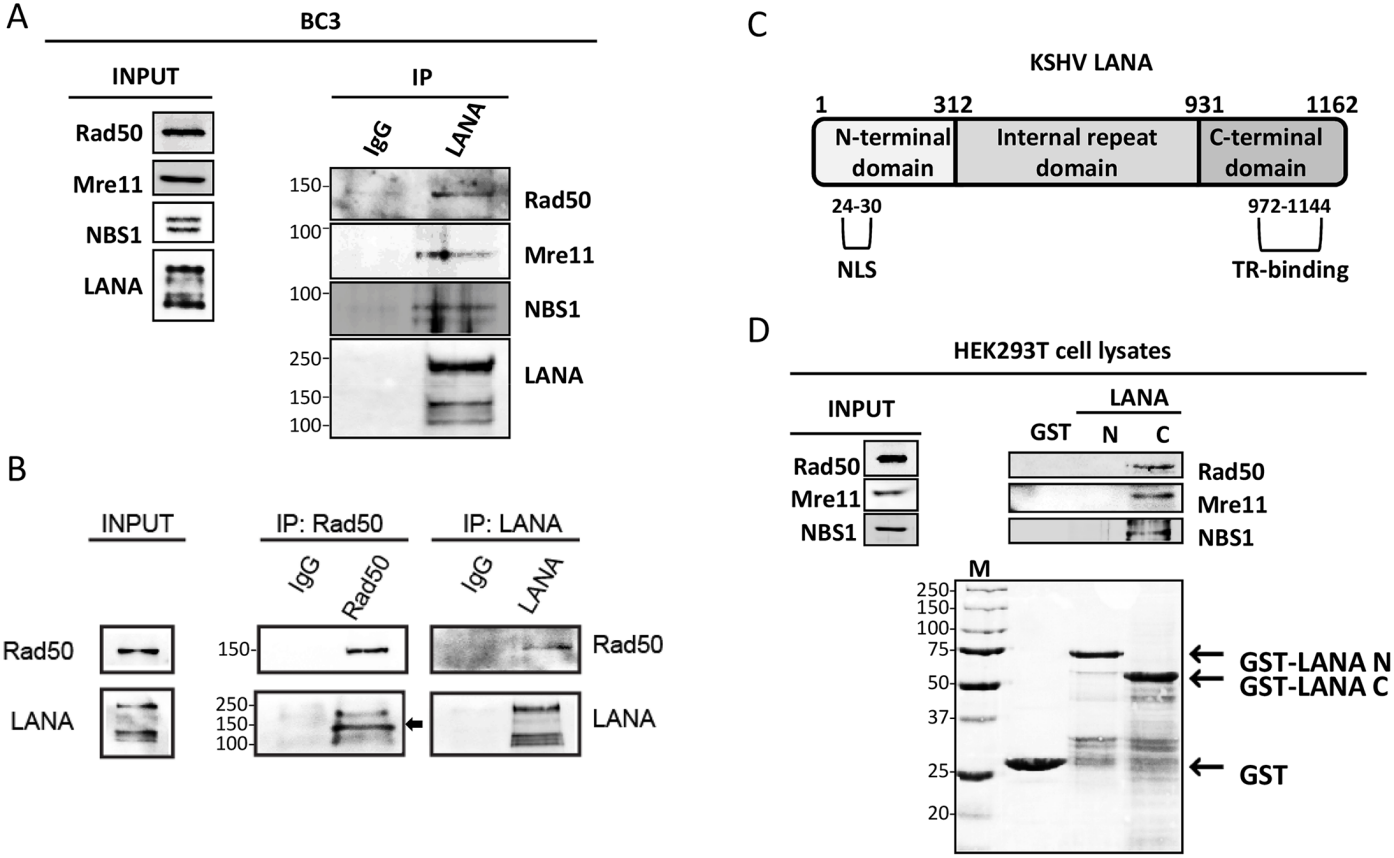
KSHV LANA recruits MRN (Mre11-Rad50-NBS1) complex. (**A**) Co-immunoprecipitation of endogenous LANA and MRN proteins in BC3 cells. Cells were lysed using TBS-T buffer and the cell lysate was incubated with benzonase. After centrifugation, supernatant was incubated overnight with anti-LANA or IgG-control beads. The precipitated complexes were analyzed for the presence of endogenous Rad50, Mre11 and NBS1 by SDS-PAGE and immunoblotting. For the input, see Materials and methods. (**B**) Co-immunoprecipitation of endogenous LANA and Rad50 in BC3 cells. Co-immunoprecipitation of endogenous Rad50 was performed and analysed as in (A), but with anti-Rad50-antibody-coated-beads (left) or anti-LANA coated beads (right). The arrowhead indicates the smaller LANA forms co-immunoprecipitating with Rad50 (see text). (**C**) Schematic representation of LANA domain structure. NLS: Nuclear Localization Signal; TR: KSHV Terminal Repeats. (**D**) Pull-down assay with GST-fused LANA-C (aa 931–1162) and LANA-N (aa 1–312) proteins and HEK293T cell lysates. HEK293T were lysed with TBS-T buffer and incubated 4 hours with GST-fused proteins or GST alone, as negative control. *Top*: immunoblot for endogenous Rad50, Mre11 and NBS1 bound to GST-fused LANA fragments. *Bottom*: Ponceau staining to detect GST-fused proteins. (M) for marker.

We confirmed the interaction of LANA with Rad50, Mre11 and NBS1 in the PEL-derived cell lines BC3 and BCBL-1, as well as in BrK.219 (a BJAB cell line stably infected with a recombinant KSHV virus [51]) by co-immunoprecipitation with anti-LANA-antibody-coupled beads and immunoblotting for Rad50, Mre11 and NBS1 (see Fig 1A and 1B for co-immunoprecipitation from BC3 cells, see S1A and S1B Fig for co-immunoprecipitation from BCBL-1 and BrK.219 cells). Before immunoprecipitation cell lysates were incubated with benzonase to digest nucleic acids and avoid DNA-mediated interactions. The interaction between LANA and Rad50 could also be shown by immuno-precipitating LANA with anti-Rad50-antibody-coupled beads and checking for LANA binding by immunoblotting (Fig 1B and S1 Fig). Both assays show that LANA interacts with the MRN complex in latently KSHV infected B cells. Interestingly, as indicated by an arrowhead in Fig 1B, smaller LANA isoforms were preferentially immunoprecipitated by Rad50 compared to those immunoprecipitated with a LANA antibody (Fig 1B). Subsequently, we investigated which region of LANA (Fig 1C) is responsible for the interaction with the MRN complex by performing GST-pull down assays with the N- (aa1-312) and C- (aa931-1162) terminal domains of LANA fused to GST (Fig 1D). HEK293 cell lysates were incubated with GST-fused LANA domains and the interaction with endogenous MRN complex proteins was analyzed by SDS PAGE and immunoblotting. The results suggest that all three MRN complex components bind to the C-terminal domain of LANA (Fig 1D). We next attempted to map the interaction site in LANA more closely by using GST-fused fragments of the LANA C-terminal domain. The results (S2 Fig) suggested that this interaction may involve multiple contact points in the LANA C-terminal region, in particular within the LANA domain binding to viral DNA (S2 Fig), the structure of which has recently been solved [19,20,22].

### KSHV LANA recruits Rad50 and Mre11 in the cytoplasm

**Fig 2:**
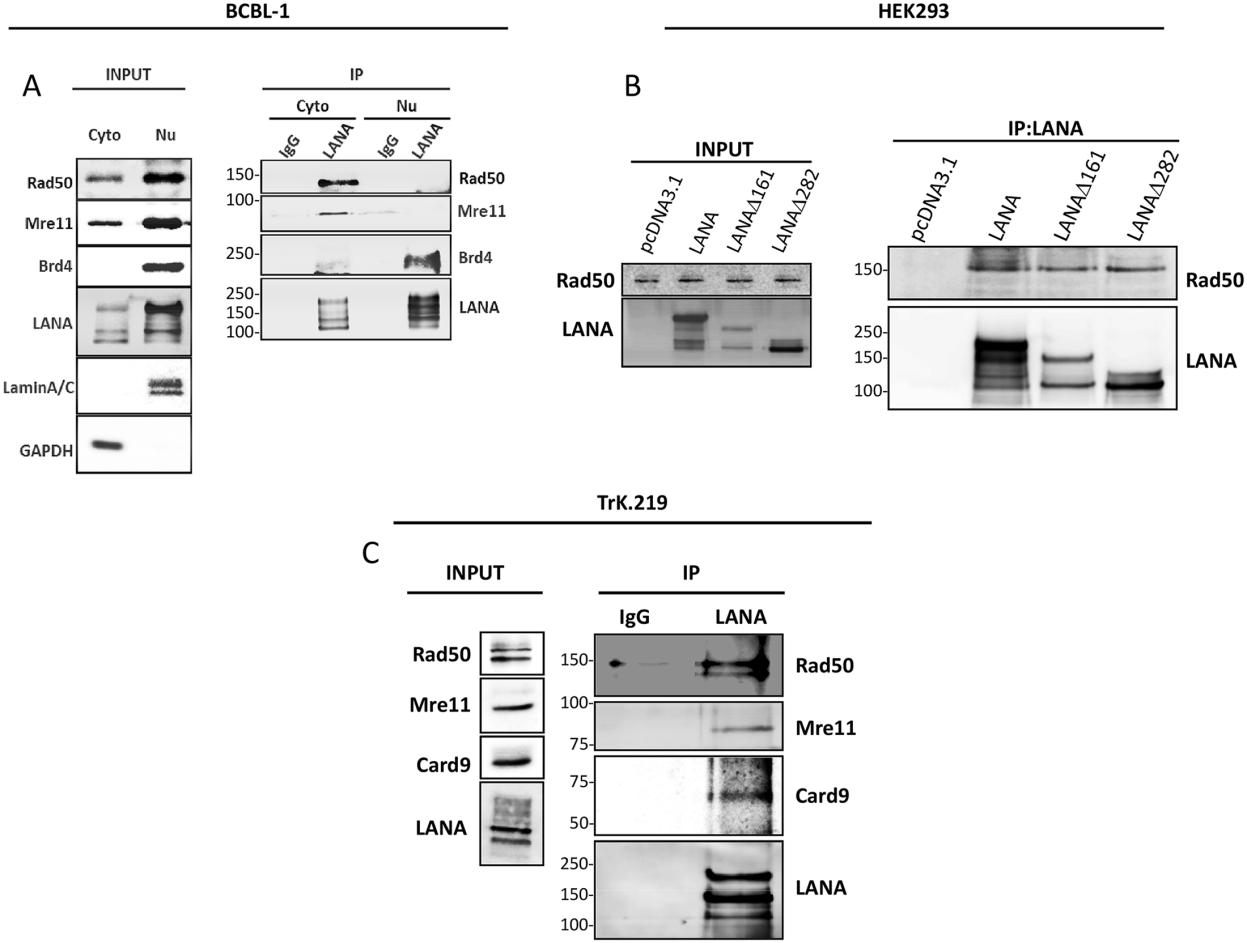
KSHV LANA recruits Rad50 and Mre11 in the cytosol. (**A**) Co-immunoprecipitation of endogenous LANA, Rad50, Mre11 and Brd4 in BCBL-1 cells upon cytosolic-nuclear fractionation. Cells were lysed and cytoplasmic extracts (Cyto) and nuclear extracts (Nu) were prepared using the Thermo-Fischer Nu-Cyto fractionation kit following the manufacturer‘s instructions. Cytoplasmic and nuclear fractions were incubated overnight with sepharose beads coated with LANA-antibody or IgG-control. Left (INPUT, see Materials and methods): Brd4, Lamin A/C and GAPDH immunoblots were analyzed to confirm the efficiency of the fractionation. Right (IP): immunoprecipitation with LANA-antibody or IgG-control coated-beads and immunoblot for endogenous Rad50, Mre11 and Brd4. (**B**) Co-immunoprecipitation of endogenous Rad50 and full-length LANA or ΔN mutants (Δ161 and Δ282) transfected into HEK293 cells. HEK293 cells were transfected with LANA constructs (or empty vector). 48 hours later cells were lysed and incubated with benzonase. After centrifugation, cells were incubated overnight with beads coated with LANA-antibody. Left (INPUT): immunoblot to check the expression of LANA constructs and the endogenous Rad50 in the cells. Right (IP from LANA-antibody-coated-beads): immunoblot for endogenous Rad50 co-immunoprecipitation. (**C**) Co-immunoprecipitation of endogenous LANA and Rad50, Mre11 and CARD9 in latently KSHV-infected THP-1 cells (TrK.219 cells, see Materials and methods). Cells were lysed and incubated with benzonase. After centrifugation, whole cell lysates were incubated overnight with beads coated with anti-LANA or IgG-control antibody. Precipitated complexes were analysed by SDS-PAGE and immunoblotting with the indicated antibodies.

According to recent findings, Rad50, together with Mre11, can translocate to the cytoplasm to sense cytoplasmic viral DNA and thereby mediate CARD9-dependent NF-κB activation [42]. The NF-κB cascade is considered to play an essential role in the maintenance of KSHV latency and also in the pathogenesis of KSHV-related diseases [52–56]. Prompted by the observation that Rad50 is associated with smaller isoforms of LANA (Fig 1B), which are known to occur in the cytoplasm [25,27], we investigated whether cytoplasmic forms of LANA interact with Rad50 and Mre11. To that end we performed co-immunoprecipitation assays, in which nuclear and cytosolic fractions from BCBL-1 cells were separated and incubated with anti-LANA-beads or IgG-control beads. We found that LANA recruits Rad50 and Mre11 mainly in the cytoplasm (Fig 2A). As a control, we also probed LANA immuno-precipitates with an antibody to Brd4, a nuclear protein known to be associated with LANA [19,24,57]. As expected, LANA and Brd4 interact in the nucleus, indicating that the buffer conditions in the nuclear extracts used for this experiment did not interfere with the interaction of LANA with its nuclear binding partners (Fig 2A). This experiment also showed that lower molecular weight forms of LANA are found predominantly in the cytoplasm (Fig 2A). To confirm these findings, we performed additional co-immunoprecipitation assays using HEK293 cells transiently transfected with constructs expressing full-length LANA or LANA ΔN mutants (Δ161 and Δ282), which lack the NLS and are therefore mainly located in the cytoplasm [25,27]. Benzonase-treated cell lysates were incubated with anti-LANA-antibody-coupled beads and the interaction with endogenous Rad50 was analyzed by immunoblotting (Fig 2B). Our results confirm that Rad50 can be recruited by both full-length LANA as well as cytosolic LANA ΔN isoforms (Fig 2B).

Our observation that cytoplasmic LANA variants recruit Rad50/Mre11 in the cytoplasm could suggest that cytoplasmic LANA might modulate the recently described CARD9-dependent activation of NF-κB, triggered as a result of cytoplasmic DNA sensed by Rad50 [42]. Since this pathway was shown to operate in myeloid cells [42], we stably infected a human leukemia monocytic cell line (THP-1) with a recombinant KSHV virus (TrK.219, see Materials and methods) and used it to confirm the interaction between LANA and the Rad50-Mre11-CARD9 DNA sensor complex (Fig 2C). Cells were lysed and incubated first with benzonase, then with anti-LANA-coupled (or IgG-coupled) beads. The interactions were analyzed by immunoblotting. Our results (Fig 2C) show that LANA recruits all the cellular proteins (Rad50, Mre11 and CARD9) recently shown to be involved in the sensing of cytosolic viral DNA and the downstream activation of the canonical NF-κB pathway [42].

### A cytoplasmic form of LANA is involved in the modulation of the canonical NF-κB pathway

**Fig 3:**
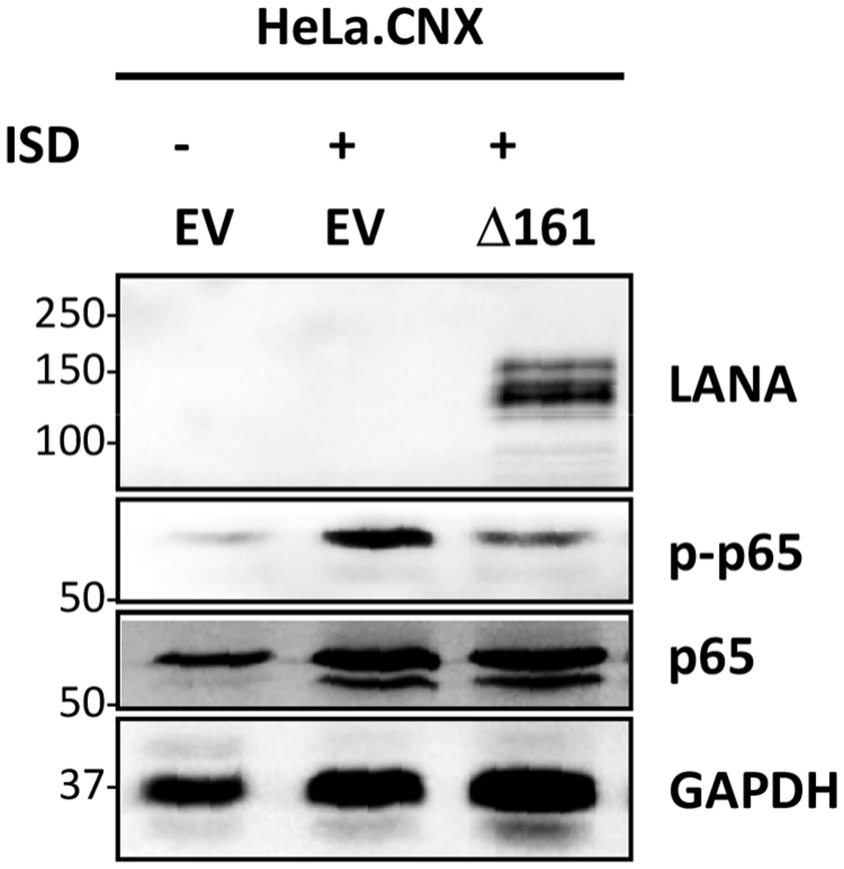
LANA Δ161 modulates the activation of NF-kB cascade triggered by cytosolic DNA. HeLa.CNX cells were transfected with the LANA Δ161 plasmid (or empty vector, EV) for about 48 hours. Cells were then stimulated with ISD (4μg/well) using Lipofectamine2000 following the manufacturer‘s instructions for 6 hours. Afterwards cells were lysed with TBS-T buffer and phosphorylation levels of p65 were analyzed by immunoblotting.

In order to determine whether cytosolic LANA isoforms can modulate the activation of NF-κB triggered by cytosolic DNA, we transiently transfected HeLa cells (Fig 3 and S3 Fig) with the construct expressing an NH_2_-terminally truncated cytoplasmic LANA isoform (LANA Δ161) [25,27] and shortly stimulated them with exogenous naked DNA (Interferon stimulatory DNA, ISD). Two different HeLa sublines, HeLa.MZ and HeLa.CNX, were chosen for this experiment (S3 Fig). Subsequently, cells were lysed and the phosphorylation of NF-κB RelA (p65), as well as the regulators of IFN-induction TBK-1 and IRF3, was analyzed by immunoblotting. As previously reported [27], HeLa.MZ cells showed an increased TBK-1 and IRF3 phosphorylation in response to ISD, reflecting an activation of the cGAS-STING cascade; this activation was inhibited by the cytoplasmic LANA Δ161 isoform (S3 Fig), consistent with previously published data [27]. In contrast, HeLa.CNX cells showed no phosphorylation of IRF3 in response to ISD stimulation, suggesting that this pathway is not fully active in this cell line (S3 Fig). However, ISD stimulation induced phosphorylation of p65/RelA in HeLa.CNX cells, and this activation of the NF-κB pathway could be inhibited by transfecting LANA Δ161 (Fig 3 and S3 Fig). This cytoplasmic isoform also inhibited p65/RelA phosphorylation in HeLa.MZ (S3 Fig). As the cGAS-TBK1-IRF3 signaling axis was deficient in HeLa.CNX, we established a HeLa.CNX cell line which was stably infected with KSHV (see Materials and methods) to study the canonical NF-κB modulation by truncated LANA and the MRN complex in the context of KSHV latent infection and in the absence of cGAS-induced IFN activation.

### Silencing of Mre11 and inhibition of the NF-κB pathway by cytoplasmic LANA promotes KSHV lytic reactivation

**Fig 4:**
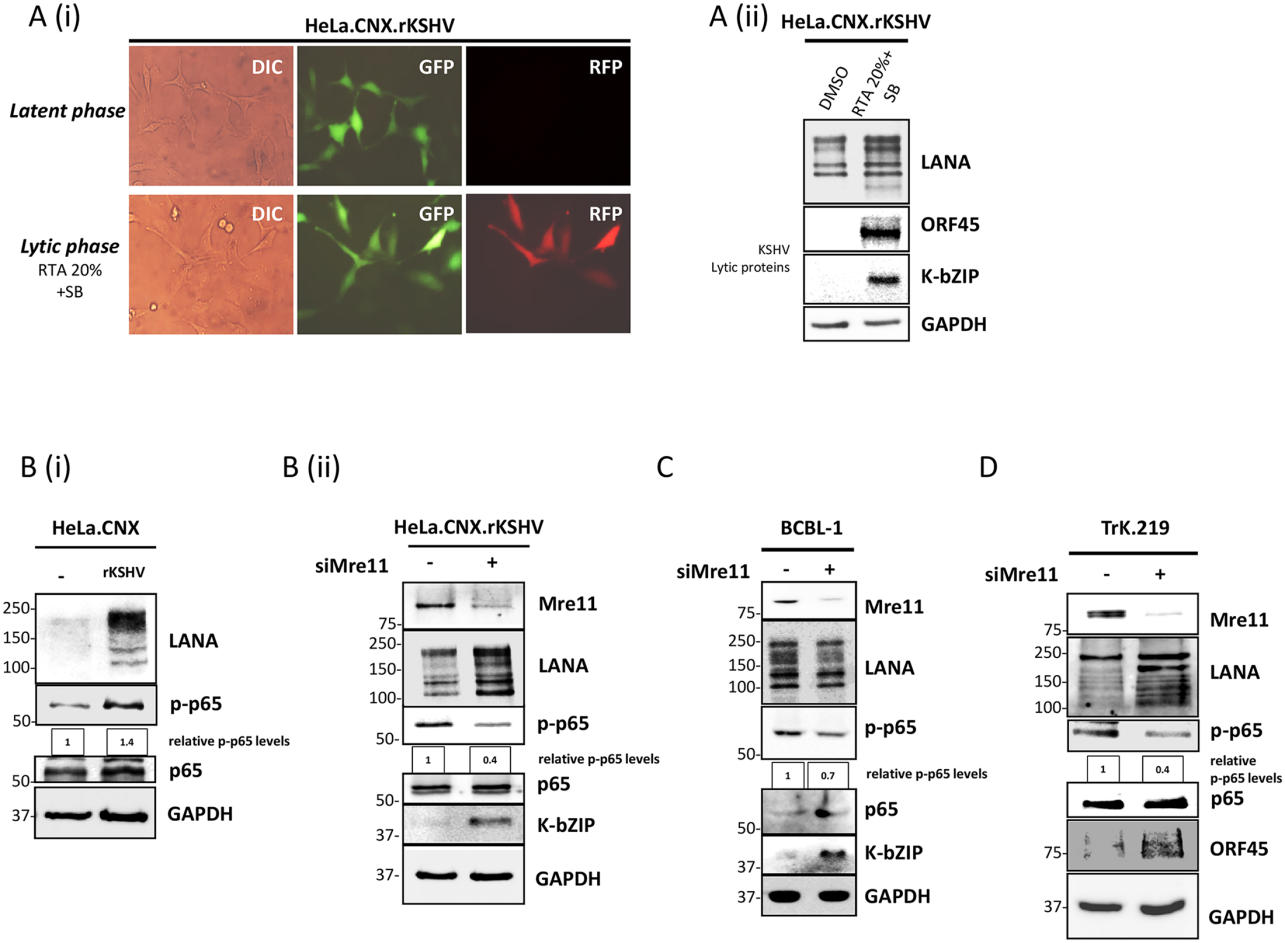
Inhibition of p65 phosphorylation and KSHV lytic reactivation upon Mre11 silencing in KSHV infected cell lines. (**A**) HeLa.CNX cells were stably infected with rKSHV.219 (see Materials and methods) and later reactivated using 20% RTA (vol/vol, see Materials and methods) and sodium butyrate (1.5 mM) for 24 hours: (**i**) RFP signal and (**ii**) ORF45 and K-bZIP expression were analyzed to confirm the KSHV lytic reactivation in these cells. (**B**) HeLa.CNX.rKSHV cells were transfected with siRNA against Mre11 (pool of 3 siRNA sequences) or negative control siRNA (see Materials and methods) for 48 hours. Cells were lysed with TBS-T buffer and protein levels analyzed by immunoblotting. (**C**-**D**) Downmodulation of p-p65 level and KSHV lytic reactivation upon Mre11 silencing, by siRNA transfection in BCBL-1 (**C**) and in TrK.219 (**D**) cells. Cells were microporated (see Materials and methods) with siRNA (300pmol/well) against Mre11 (pool of 3 siRNA sequences) or non targetting siRNA (as negative control). After 2 days cells were lysed with TBS-T buffer and KSHV K-bZIP or ORF45 and p-p65 levels were analyzed by immunoblotting. Phospho-p65 levels in Fig 5B–5D were digitally quantified (see Materials and methods).

Latently KSHV-infected HeLa.CNX cells (HeLa.CNX.rKSHV) were treated with a recombinant baculovirus expressing the regulator of the lytic replication cycle, RTA [58], and sodium butyrate [27,32,53,59,60] to induce the lytic phase and thereby confirm that KSHV could be reactivated in this cell line (Fig 4Ai and 4Aii). Following the treatment with 20% RTA (vol/vol, see Materials and methods) and 1.5 mM sodium butyrate for 24 hours, the expression of the RFP lytic reporter in the recombinant KSHV.219 virus [51,58] used for these experiments was switched on (Fig 4Ai) and the early KSHV protein K-bZIP and the Orf45-encoded tegument protein were expressed (Fig 4Aii). HeLa.CNX.rKSHV cells have much higher levels of phosphorylated p65/RelA than uninfected HeLa.CNX cells (Figs 4Bi, 4Bii and 5A), in line with the known ability of several latent KSHV proteins such as vFLIP and LANA to activate the NF-κB pathway [52,53,61]. To assess the role of the MRN complex in NF-κB activation and in KSHV lytic reactivation, we inhibited Mre11 expression by siRNA transfection in HeLa.CNX.rKSHV cells (using a pool of three siRNAs, Fig 4Bii, or the same three siRNAs transfected individually, S4 Fig). In these cells Mre11 silencing triggers KSHV lytic reactivation, as indicated by an increase in K-bZIP levels and a reduction in the levels of phosphorylated p65/RelA (Fig 4Bii). We next explored if this contribution of Mre11 to the maintenance of KSHV latency also applied to other KSHV-infected cell lineages. Similar to the results obtained in KSHV-infected HeLa cells, we found that silencing of Mre11 in the PEL cell line BCBL-1 as well as in the KSHV-infected THP-1 cell line TrK.219, resulted in KSHV reactivation from latency, as indicated by increased levels of, respectively, K-bZIP or ORF45, along with a decrease in p65/RelA phosphorylation (Fig 4C and 4D). Together, these results indicate that in these KSHV-infected cells Mre11 contributes to the activation of the NF-κB pathway that promotes KSHV latency [14,56]. We could not achieve an efficient silencing of Rad50 in PEL cells (or any other latently KSHV-infected cell lines), and therefore we were not able to assess if Rad50 contributes to the inhibition of the lytic cycle in a way similar to Mre11.

**Fig 5:**
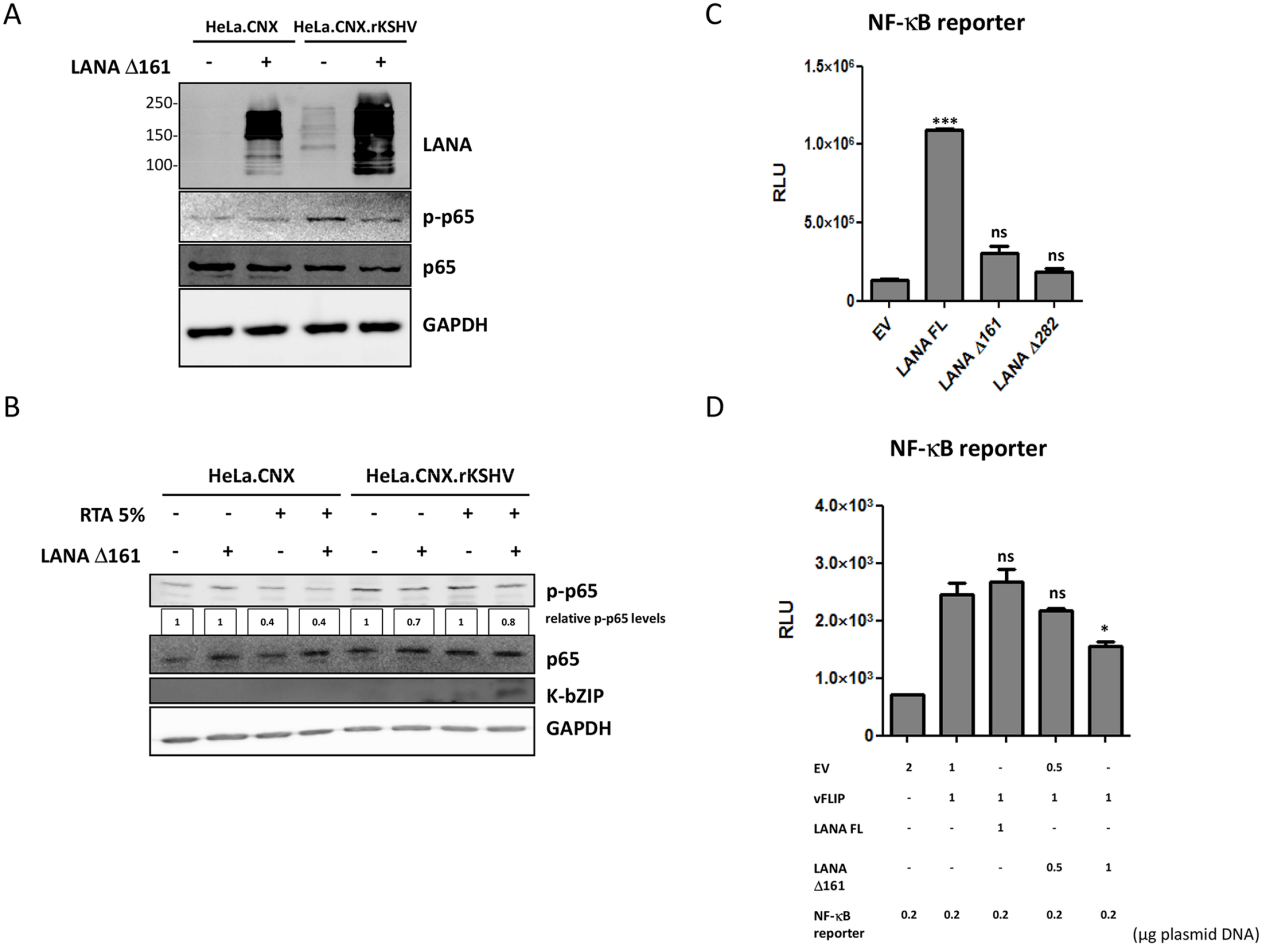
Inhibition of canonical NF-κB cascade and KSHV lytic reactivation upon LANA Δ161 overexpression. (**A-B**) HeLa.CNX.rKSHV and the parental HeLa.CNX cells were transfected with LANA Δ161 plasmid or empty vector for 48 hours and (**B**) treated with 5% RTA (vol/vol, see Materials and methods) for about 24 hours. Cells were lysed with TBS-T buffer and lysates resolved by immunoblotting. Phospho-p65 levels were digitally quantified (see Materials and methods). (**C**) HEK293 cells were transfected with the plasmid expressing full-length LANA, the truncated LANA isoforms or the empty vector (2 μg/well), together with an NF-κB reporter vector (200 ng/well). The luciferase activity was measured 48 hours later in duplicates and the statistical analysis was performed with two-tailed student’s t-test. Statistical significance of the difference between control (pcDNA3.1) and LANA (FL or Δ161 or Δ282) transfected samples is shown: (***) for p<0.005 and (ns) for not significant. (**D**) HeLa.CNX cells were transfected with the plasmid expressing vFLIP or the empty vector (1μg) and additionally with the plasmid expressing full-length LANA (1μg) or the truncated isoform (Δ161, 0.5–1μg) or the empty vector (2μg of plasmid DNA in total per condition), together with NF-κB reporter vector (200 ng/well). The luciferase activity was measured 48 hours later in duplicates and the statistical analysis was performed with two-tailed student’s t-test. Statistical significance of the difference between vFLIP alone and vFLIP-LANA (FL or Δ161) transfected samples is shown: (*) for p<0,05 and (ns) for not significant.

To explore if cytoplasmic LANA could modulate NF-κB via Mre11 and thereby affect lytic reactivation, infected and uninfected HeLa.CNX cells were transfected with LANA Δ161 or the empty vector and p65/RelA phosphorylation was analyzed by immunoblotting (Fig 5A). Our results show that LANA Δ161 overexpression reduces p65/RelA phosphorylation level in HeLa.CNX.rKSHV cells (Fig 5A). Furthermore, HeLa cells were treated with low amounts of RTA (5% vol/vol, Fig 5B) to induce the lytic reactivation only at a minimal level and were additionally transfected with LANA Δ161 or the empty vector. Our results show that the LANA Δ161 overexpression supports the lytic reactivation in HeLa.CNX.rKSHV cells induced by low levels of RTA, as highlighted by increased levels of K-bZIP expression (Fig 5B). In addition, levels of phosphorylated p65/RelA were reduced following transfection of LANA Δ161 and upon lytic reactivation indicating an antagonistic role of truncated LANA for canonical NF-κB activation (Fig 5A and 5B). In addition, the co-expression of Mre11 together with Δ161 LANA counteracts the Δ161 LANA-mediated downmodulation of p-p65 levels (S5 Fig). To explore the role of NH_2_-terminally truncated cytoplasmic LANA variants further, we compared the ability of full-length LANA, LANA Δ161 and LANA Δ282 to activate an NF-κB dependent reporter vector in HEK293 cells (Fig 5C). As previously reported [61], full-length LANA was found to activate NF-κB-dependent transcription (Fig 5C). In contrast, LANA Δ161 and LANA Δ282 failed to do so (Fig 5C). However, when we explored the ability of LANA Δ161 to modulate the activation of the NF-κB pathway by the potent NF-κB activator and IKKγ ligand vFLIP [53,62–65], we found that LANA Δ161 could inhibit vFLIP-induced NF-κB activation in a dose-dependent manner, while full-length LANA could not (Fig 5D).

**Fig 6:**
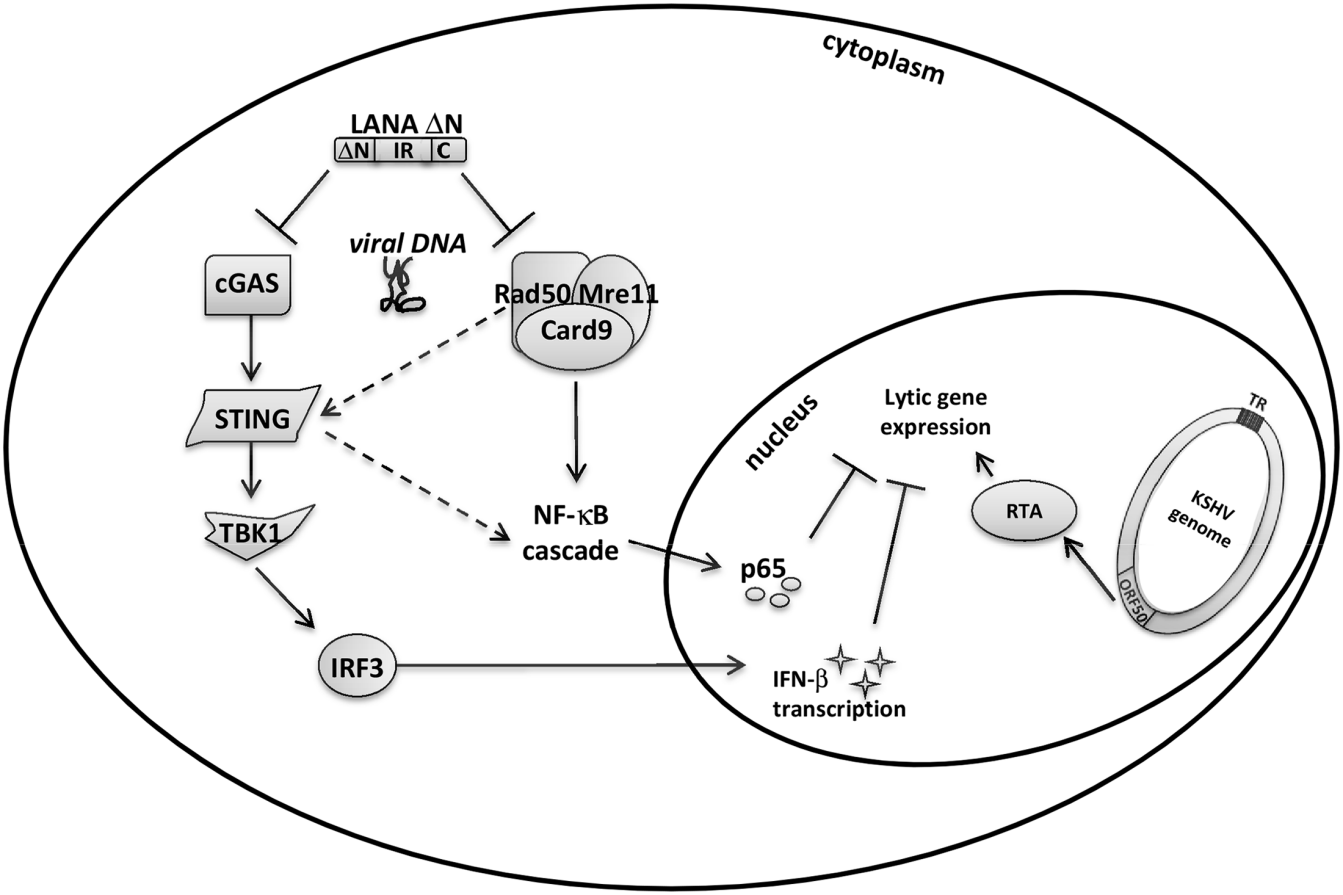
Model of LANA antagonizing cytoplasmic DNA sensors. Cytoplasmic KSHV LANA isoforms recruit and antagonize cellular DNA sensor proteins cGAS as well as the Rad50-Mre11-CARD9 complex to inhibit innate immunity responses (IFN-β and NF-κB) and support KSHV lytic reactivation from latency. During KSHV lytic reactivation, free viral DNA in the cytosol is detected by host DNA sensors, such as cGAS and the Rad50/Mre11/CARD9 complex. The cGAS-STING cascade leads to IFN-β production, whereas the Rad50/Mre11/CARD9 complex is responsible for NF-κB cascade activation. A cross-talk between these two pathways may also be possible as indicated by the dashed arrows (Fig 6). Triggering of the Rad50/Mre11/CARD9 complex leads to the activation and nuclear accumulation of NF-κB p65 and the subsequent production of chemokines and cytokines. These events would interfere with the efficient KSHV lytic replication and therefore KSHV LANA ΔN isoforms block these signalling cascades by recruiting and inhibiting the upstream activators (cGAS as well as Rad50/Mre11).

Taken together, our results suggest that cytoplasmic forms of LANA may target Rad50 and Mre11, and thereby antagonize the activation of NF-κB and NF-κB-dependent suppression of the KSHV lytic cycle (Fig 6).

## Discussion

An involvement of some DDR proteins in the innate immune response is increasingly appreciated [42–44]. This highlights the similarities between the recognition of host DNA damage for subsequent repair, and of foreign DNA for the purpose of triggering an innate immune response leading to the activation of type I interferon and NF-κB-dependent pathways. In particular, components of the MRN DSBs repair complex, Rad50 and Mre11, have recently been shown to sense cytoplasmic “foreign” DNA and to activate the NF-κB pathway in a CARD9-dependent manner [42].

In the present study, we found that LANA recruits Rad50 and Mre11 mostly in the cytosol of naturally KSHV-infected B cells (Fig 2A), that a cytoplasmic form of LANA may antagonize the activation of NF-κB induced by transfected DNA (Fig 3) or vFLIP (Fig 5C) and that silencing of Mre11 promotes KSHV lytic replication in parallel to reduced NF-κB p65 phosphorylation (Fig 4Bii). These results are in line with the newly described function of DDR proteins in the context of cytosolic DNA sensing and inflammasome response [42]. In contrast to full-length nuclear LANA, which is found in the characteristic nuclear speckles [8,9], cytoplasmic LANA shows a diffuse distribution [25,27]. This absence of any cytosolic LANA-containing structure prevented us from showing a LANA-Mre11/Rad50/CARD9 co-localization in the cytoplasm of infected cells and we therefore had to rely on co-immunprecipitation experiments from cytosolic fractions as shown in Fig 2A. We observed the interaction of cytoplasmic LANA with Mre11 and Rad50 in cells without a detectable CARD9 expression (e.g. BCBL-1, BJAB.rKSHV) and therefore believe that it is unlikely that CARD9 is responsible for bridging Mre11 and Rad50 to cytoplasmic LANA. However, we cannot formally exclude this possibility.

We have previously reported that cytoplasmic forms of LANA can promote lytic reactivation by antagonizing another cytoplasmic DNA sensor, cGAS [27]. To discriminate between the effect of cGAS-dependent interferon induction and MRN-dependent NF-κB activation on KSHV latency or reactivation, we took advantage of the fact that the HeLa.CNX subline appears to be deficient for cGAS-dependent IRF3 phosphorylation (S3 Fig). In this somewhat artificial experimental setting, we can therefore demonstrate that cytoplasmic LANA isoforms can promote KSHV reactivation by repressing NF-κB activation.

Taken together, our observations therefore suggest that cytoplasmic forms of LANA antagonize not only cGAS-dependent type I interferon responses but also the Rad50-Mre11-CARD9-dependent activation of NF-κB pathway in response to cytoplasmic DNA (Fig 6), which is present during herpesviral lytic replication [27,42,66,67]. The fact that cytoplasmic LANA appears able to neutralize both these pathways testifies to their importance in restricting “lytic”, productive, KSHV replication. The NF-κB pathway has previously been shown to be required for maintaining the latency of γ2-herpesviruses [14,56], and the KSHV vFLIP protein, known to activate both NF-κB and the expression of interferon-dependent cellular genes, also contributes to the maintenance of KSHV latency [53,55,68–71]. This is supported by the observation shown in Figs 4Bi and 5A that KSHV-infected HeLa cells display higher levels of NF-κB p65 phosphorylation than uninfected controls. It is thus conceivable that KSHV needs to counteract both these restrictive pathways to successfully reactivate from latency. This may also be necessary as a cross-talk between these two pathways (cGAS-STING activating NF-κB and *vice versa*) may be possible [44]. Cytoplasmic isoforms of LANA, which lack the NLS-containing N-terminal region, have been shown to be more strongly expressed during lytic reactivation [27] and may result from the use of alternative in-frame translational start codons [25] or the cleavage of an N-terminal LANA fragment by Caspase 3 [72]. Together with our previous report [27], our recent findings may therefore indicate a role for cytoplasmic LANA isoforms as viral antagonists of the innate immune response. Furthermore, our observations (Fig 5C and 5D) indicate that cytoplasmic LANA isoforms may act as antagonists of full-length, nuclear LANA, at least with regard to antagonizing the activation of the NF-κB pathway, which is thought to contribute to the establishment and/or maintenance of latency [52,54,61]. Cytoplasmic LANA would thus support the action of the lytic switch protein, RTA, encoded by ORF50, which has been shown to counteract vFLIP-dependent NF-κB activation and its contribution to the maintenance of latency by aiding the degradation of vFLIP by the proteasome [53,71]. Taken together, our results suggest a role for cytoplasmic LANA variants in modulating NF-κB activity by recruiting components of the MRN DNA repair complex and thereby regulating KSHV latency.

## Materials and methods

### Cell culture

HEK293 (ATCC CRL-1573), HEK293T (ACC 305 from the German Collection of Microorganisms and Cell Cultures-DMSZ), HeLa.MZ (provided by Marino Zerial, Max Plank Institute of Cell Biology and Genetics, Dresden) and HeLa.CNX (provided by Beate Sodeik, Hannover Medical School, Hannover) cells were cultured in Dulbecco’s modified Eagle medium (DMEM, containing D-glucose, L-glutamine, pyruvate) supplemented with 10% fetal calf serum (FCS). KSHV-infected PEL-derived B cell lines (BC3, BCBL-1), the B cell line BJAB (ACC-757 from the German Collection of Microorganisms and Cell Cultures-DMSZ) stably infected with recombinant KSHV (BrK.219) [51,73] and the human leukemia monocytic cell line THP-1 (ACC-16 from the German Collection of Microorganisms and Cell Cultures-DMSZ) stably infected with rKSHV.219 (TrK.219) were grown in RPMI medium 1640 (containing L-glutamine) supplemented with 20% FCS, and in case of BrK.219 and TrK.219 with 4 μg/mL puromycin (Sigma, P8833). Cells were grown at 37°C in a 5% CO_2_ incubator. Adherent cells were plated in 6-well plates 24 hours before transfection (5x10^5^ cells per well), or were microporated (1x10^6^ cells per well, in 12-well plates). The suspension cells were split at a ratio 1:2 one day before microporation (1x10^6^ cells per condition) or lysed for binding assays (1x10^7^ cells per condition). HeLa.CNX cells were latently infected with a recombinant KSHV virus containing a puromycin-resistance cassette, which had been produced using BrK.219 cells. Briefly, BrK.219 cells were stimulated with α-IgM (2.5 μL/mL) for 48 hours. After centrifugation, supernatant, containing infectious virions, was collected and filtered using a 0.45μm pore-size filter to remove cell debris and stored at +4°C. HeLa.CNX cells were seeded in a 12-well plate and one day later infected at an MOI of 10 with BrK.219-derived virus. After 48 hours, puromycin (1 μg/mL) was added to the medium for selection of the KSHV-infected (+) HeLa cells. Three weeks later, the stably KSHV infected cell line was tested for viral proteins expression (by immunoblotting). The TrK.219 cell line was established by infecting THP-1 cells with rKSHV.219 at an MOI of 10. Puromycin was added to the medium for selection at final concentration of 4 μg/mL. After four weeks, KSHV stably infected THP-1 cells (TrK.219) were tested by immunoblotting and PCR.

### KSHV lytic cycle induction

KSHV lytic reactivation was induced as followed: HeLa.rKSHV cells were treated with a combination of RTA, ectopically expressed from a baculoviral vector (calculated as volume of medium containing baculovirus / volume of total medium in one well, vol/vol), and Sodium Butyrate (see figure legends for further details). Cell pictures to check for GFP and RFP expression were taken using a Nikon Intensilight C-HGFI microscope.

### Reagents and plasmids

Full-length LANA was expressed from a vector with the pcDNA3.1 backbone. Human Mre11 was expressed by transfecting a plasmid purchased from Addgene (plasmid # 82033) and the corresponding empty vector (plasmid # 46960) was used as a control. Adherent cells were transfected using Fugene6 (Promega, E269A) according to the manufacturer’s instructions. Cells were stimulated with naked DNA (ISD Naked, InvivoGen, tlrl-isdn) by transfection with Lipofectamine2000 (Invitrogen by Life Technologies, 11668–027), using the conditions indicated in the figure legends. siRNAs were purchased from Dharmacon: human Mre11 custom siRNA pool (#1: ccugccucgaguuauuaaguu; #2: cugcgaguggacuauaguguu; #3 gaugccauugaggaauuaguu), siGENOME Non-Targeting Pool#2 (D-001206-14-50). siRNAs were prepared according to the manufacturer’s instructions and transfected at the concentrations indicated in the figure legends using the Neon transfection system (Thermo-Fischer Scientific) under the following microporation conditions: 1150V, 30ms, 2 pulses.

### Cytosolic/nuclear fractionation

Cytosolic/nuclear fractions were prepared from whole cell lysates using NE-PER nuclear and cytoplasmic extraction reagents (ThermoFischer, 78835) according to the manufacturer’s instructions. All extracts were incubated immediately with LANA-beads or stored at -80°C.

### Binding assays

Production of GST fusion proteins and GST-pulldown assays were performed as previously described [15,16]. Endogenous co-immunoprecipitation assays were performed using PEL cell lines (1x10^7 cells/condition) harvested with TBS-T buffer (20 mM TRIS-HCl pH 7.4, 150 mM NaCl, 50 mM MgCl_2_, 1% TritonX-100). Benzonase nuclease (Merck Millipore, 71205–3) was added to whole cell lysates (50U each 2x10^6^ cells) for 30 minutes at RT to digest nucleic acids. Subsequently, the samples were centrifuged at 20800 g for 10 minutes at +4°C and the supernatants used for immunoprecipitation. The input control corresponds to 4% of the lysate used for an individual immunoprecipitation sample. Protein G sepharose beads (GE Healthcare) were washed with TBS-T buffer and incubated for 5 hours at +4°C with α-LANA (rat, from ABI, 18-210-100) or α-Rad50 (mouse, from GeneTex, GTX70228) or negative control (α-IgG rat or α-IgG mouse) antibody. Finally, antibody-coupled-beads were washed with TBS-T buffer, resuspended in PBS and used immediately or stored shortly at +4°C.

### SDS-PAGE and immunoblotting

Cell lysates, after boiling for 5 minutes at 95°C and centrifugation for 10 minutes at 20800 g, were subjected to SDS-PAGE. Proteins were detected by Ponceau S or immunoblotting, using the following primary antibodies: α-LANA (18-210-100, ABI); α-Rad50 (GTX70228, GeneTex); α-Mre11 (ab33125, Abcam); α-NBS1 (NB100-143, Novus Biologicals); α-GAPDH (14C10, Cell Signalling); α-KbZIP (F33P1, Santa Cruz Biotechnology); α-HHV-8 ORF45 (2D4A5, Santa Cruz Biotechnology); α-HHV-8 ORF57 (LS-C60137, LSBio); α-CARD9 (Cell Signalling, 12416S); α-p65 (sc-109, Santa Cruz); α-p-p65 (S536, Cell Signalling); α-Brd4 (A301-985A100, Bethyl); α-LaminA/C (sc-6215, Santa Cruz); α-IRF3 (sc-9082, Santa Cruz); α-p-IRF3 (4947S, Cell Signalling); α-p-TBK1 (3504S, Cell Signalling). Subsequently, membranes were incubated with the following secondary antibodies: α-mouse (P0260, Dako); α-rabbit (P0488, Dako); α-rat (P0450, Dako). Phospho-p65 levels were digitally quantified using ImageJ and normalized to the corresponding total p65 protein levels and the sample used as negative control.

### Luciferase reporter assay

For luciferase reporter assays, HEK293 cells were transiently co-transfected in duplicates with NF-κB reporter plasmid and expression constructs as reported in the figures legends. At the indicated time points, cells were washed once with PBS and lysed using 125μl per well of Reporter Lysis Buffer (Promega). Luciferase activity was immediately measured at the luminometer (DIGENE DIAGNOSTICS, inc.) using 30μl per condition and 100μl Luciferase Buffer (40mM Tricine, pH 7.8, 10mM MgSO_4_, 0.5mM ATP, 10mM DTT, 0.5mM Coenzyme A, 0.5mM D-Luciferine). To test for statistical significance a two-tailed T-test was used.

## Supporting information

S1 Fig LANA-Rad50 interaction in KSHV-infected cells. (**A**) Co-immunoprecipitation of endogenous LANA and Rad50 in BCBL-1 cells. Cells were lysed using TBS-T buffer and the whole cell lysate was incubated with benzonase. After centrifugation, supernatants were incubated overnight with LANA-antibody (*right*) or Rad50-antibody (*left*) or corresponding IgG-control coated-beads. Precipitated complexes were analyzed by SDS-PAGE and immunoblotting with the indicated antibodies. (**B**) Co-immunoprecipitation of endogenous Rad50 and LANA in BrK.219 cells. BJAB (KSHV-) cells were used as additional negative control. Cells were lysed using TBS-T buffer and the whole cell lysate was incubated with benzonase. After centrifugation, supernatants were incubated overnight with Rad50-antibody or IgG-control coated-beads. Precipitated complexes were analyzed by SDS-PAGE and immunoblotting with the indicated antibodies.
(TIF)

S2 Fig GST-Pulldown showing the interaction of MRN components with the LANA C-terminal region (aa. 986–1100). (**A**) Schematic diagram showing fragments of the LANA C-terminal domain GST-fused proteins used for the pull-down assay. (**B**) Pull-down assay with GST-fused LANA C-terminal domain proteins (shown in (A)) with HEK293T cell lysates. Cell lysates were incubated for four hours with the described GST-fused proteins. *Top*: immunoblot for endogenous Rad50, Mre11 and NBS1. *Bottom*: Ponceau staining to detect GST-fused proteins. (M) for marker.
(TIF)

S3 Fig LANA Δ161 modulates the activation of NF-κB cascade triggered by cytosolic DNA. HeLa.MZ and HeLa.CNX cells were transfected with the plasmid expressing the full-length (FL) or truncated (Δ161) LANA or empty vector (EV) for 48 hours. Cells were then stimulated with ISD (4μg/well) using Lipofectamine2000 following the manufacturer‘s instructions for 6 hours. Afterwards cells were lysed with TBS-T buffer and phosphorylation level of TBK-1, IRF3 and p65 were analyzed by immunoblotting. Phospho-p65 levels were digitally quantified (see Materials and methods).
(TIF)

S4 Fig Mre11 silencing and KSHV lytic reactivation. (A) BCBL-1 and (B) HeLa.CNX.rKSHV cells were transfected with individual siRNAs against Mre11 (see Materials and methods) or non-targeting siRNA as a negative control. Cells were microporated (see Materials and methods) with siRNA (300pmol/well) and after 2 days cells were lysed with TBS-T buffer. The expression of KSHV lytic proteins (K-bZIP and/or ORF45) was analyzed by immunoblotting.
(TIF)

S5 Fig Mre11 overexpression counteracts LANA Δ161-induced p-p65 downmodulation. HeLa.CNX.rKSHV cells were transfected with plasmids expressing Mre11 and/or Δ161 LANA or corresponding empty vectors for 48 hours. Cells were lysed using TBS-T buffer and phosphorylation levels of p65 were analyzed by immunoblotting and digitally quantified (see Materials and methods).
(TIF)
