# Supplementary figures and images for: Kaposi Sarcoma Herpesvirus (KSHV) Latency-Associated Nuclear Antigen (LANA) recruits components of the MRN (Mre11-Rad50-NBS1) repair complex to modulate an innate immune signaling pathway and viral latency
Source: PLoS Pathog. 2017 Apr 21;13(4):e1006335. doi: 10.1371/journal.ppat.1006335 (PMC5415203; doi:10.1371/journal.ppat.1006335)

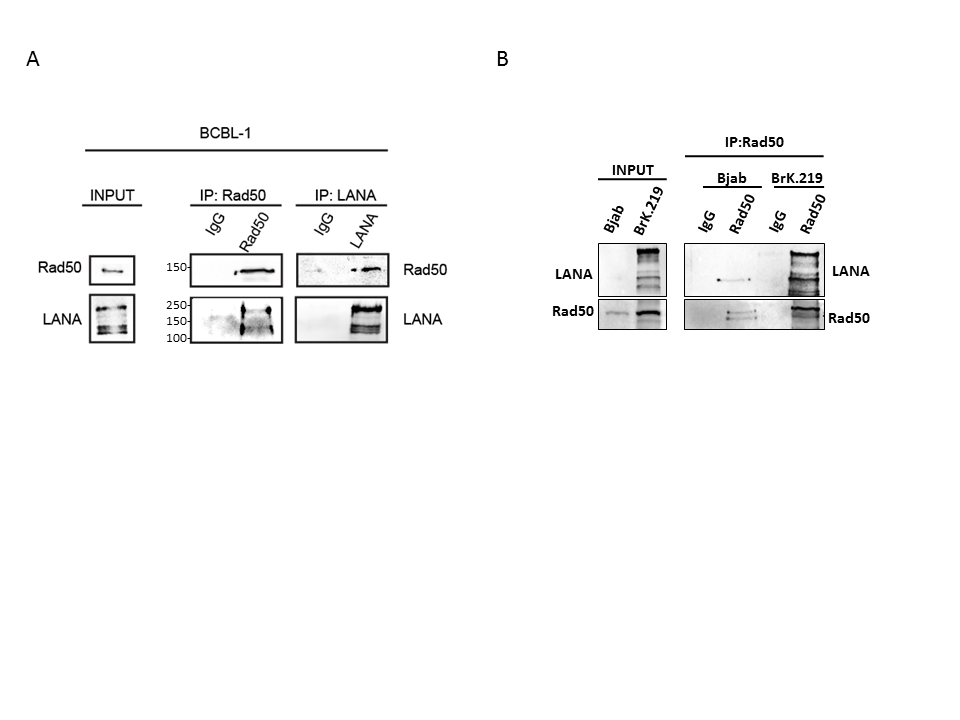

Supplement: S1 Fig — (A) Co-immunoprecipitation of endogenous LANA and Rad50 in BCBL-1 cells. Cells were lysed using TBS-T buffer and the whole cell lysate was incubated with benzonase. After centrifugation, supernatants were incubated overnight with LANA-antibody (right) or Rad50-antibody (left) or corresponding IgG-control coated-beads. Precipitated complexes were analyzed by SDS-PAGE and immunoblotting with the indicated antibodies. (B) Co-immunoprecipitation of endogenous Rad50 and LANA in BrK.219 cells. BJAB (KSHV-) cells were used as additional negative control. Cells were lysed using TBS-T buffer and the whole cell lysate was incubated with benzonase. After centrifugation, supernatants were incubated overnight with Rad50-antibody or IgG-control coated-beads. Precipitated complexes were analyzed by SDS-PAGE and immunoblotting with the indicated antibodies. (TIF) [file ppat.1006335.s001.TIF]

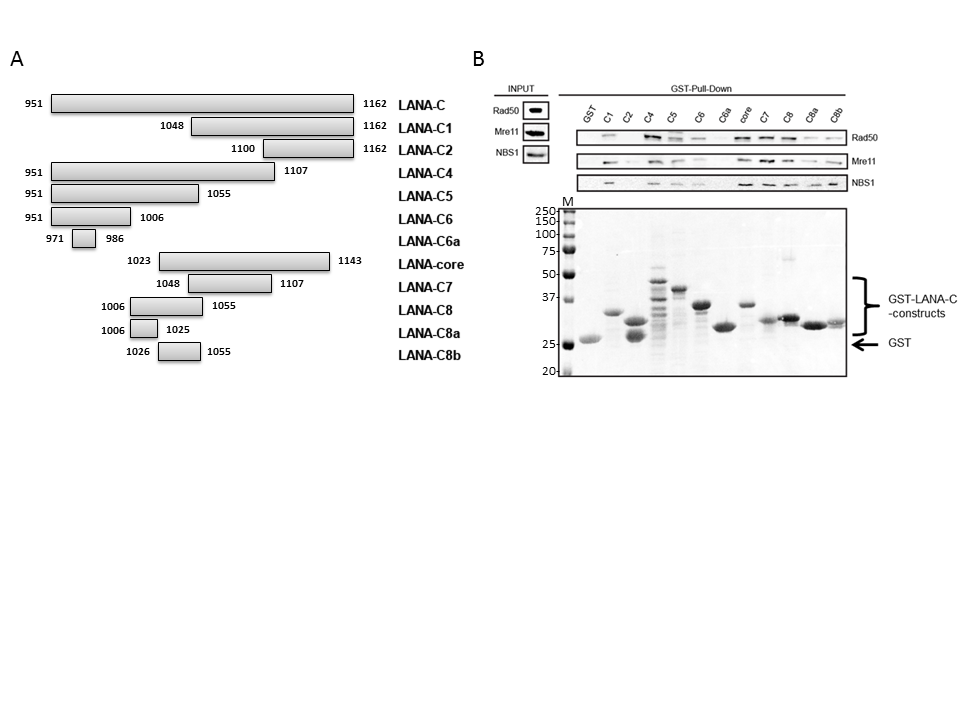

Supplement: S2 Fig — (A) Schematic diagram showing fragments of the LANA C-terminal domain GST-fused proteins used for the pull-down assay. (B) Pull-down assay with GST-fused LANA C-terminal domain proteins (shown in (A)) with HEK293T cell lysates. Cell lysates were incubated for four hours with the described GST-fused proteins. Top: immunoblot for endogenous Rad50, Mre11 and NBS1. Bottom: Ponceau staining to detect GST-fused proteins. (M) for marker. (TIF) [file ppat.1006335.s002.TIF]

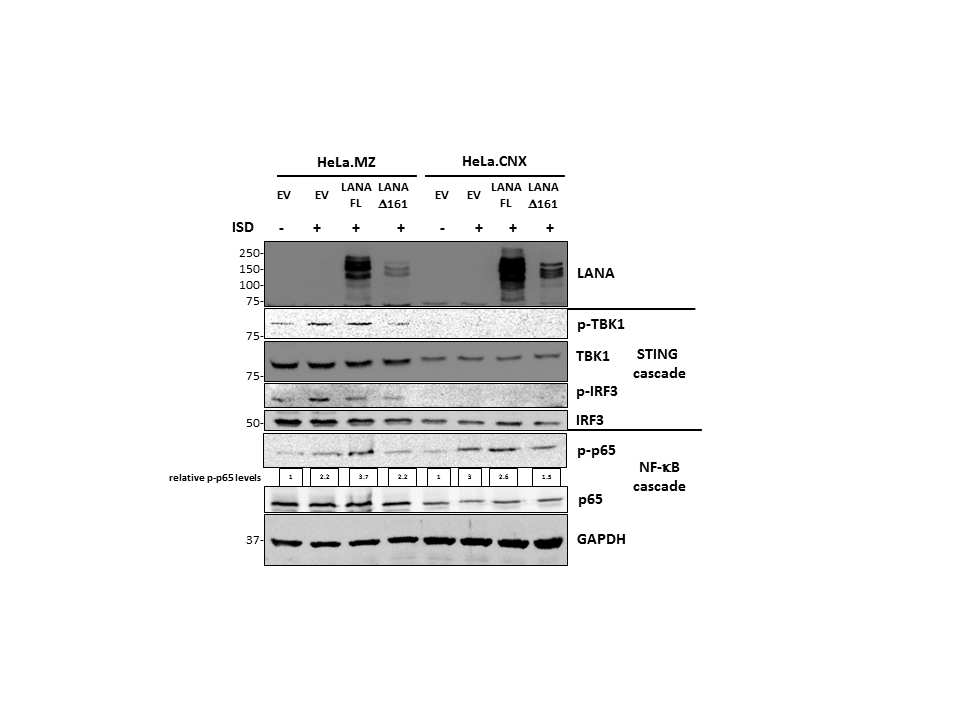

Supplement: S3 Fig — HeLa.MZ and HeLa.CNX cells were transfected with the plasmid expressing the full-length (FL) or truncated (Δ161) LANA or empty vector (EV) for 48 hours. Cells were then stimulated with ISD (4μg/well) using Lipofectamine2000 following the manufacturer‘s instructions for 6 hours. Afterwards cells were lysed with TBS-T buffer and phosphorylation level of TBK-1, IRF3 and p65 were analyzed by immunoblotting. Phospho-p65 levels were digitally quantified (see Materials and methods). (TIF) [file ppat.1006335.s003.TIF]

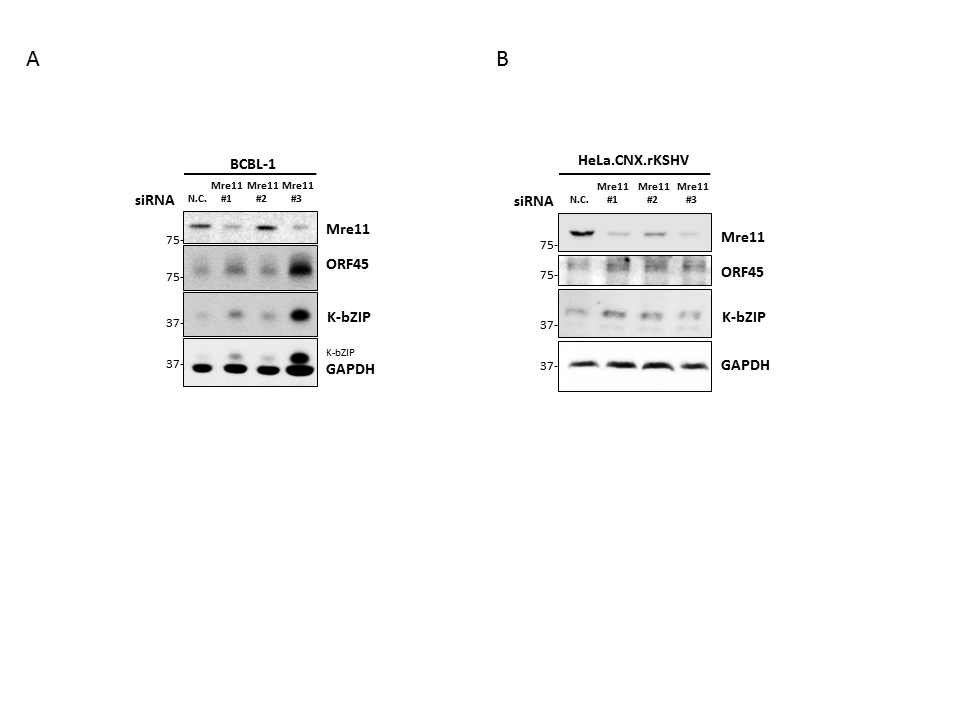

Supplement: S4 Fig — (A) BCBL-1 and (B) HeLa.CNX.rKSHV cells were transfected with individual siRNAs against Mre11 (see Materials and methods) or non-targeting siRNA as a negative control. Cells were microporated (see Materials and methods) with siRNA (300pmol/well) and after 2 days cells were lysed with TBS-T buffer. The expression of KSHV lytic proteins (K-bZIP and/or ORF45) was analyzed by immunoblotting. (TIF) [file ppat.1006335.s004.TIF]

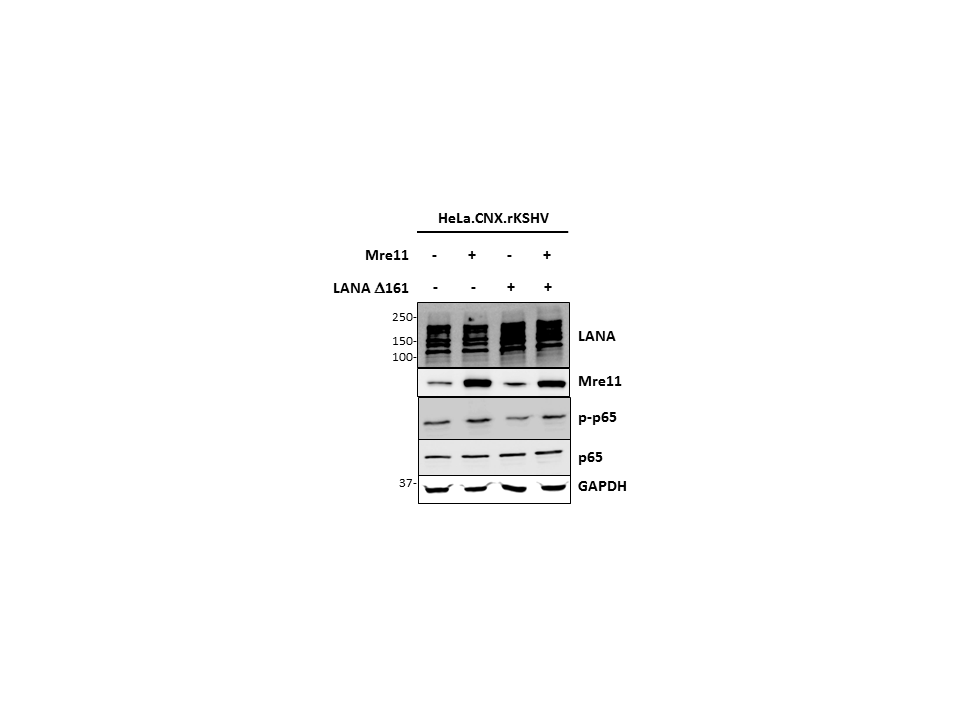

Supplement: S5 Fig — HeLa.CNX.rKSHV cells were transfected with plasmids expressing Mre11 and/or Δ161 LANA or corresponding empty vectors for 48 hours. Cells were lysed using TBS-T buffer and phosphorylation levels of p65 were analyzed by immunoblotting and digitally quantified (see Materials and methods). (TIF) [file ppat.1006335.s005.TIF]
